# Idiopathic CD4 Lymphocytopenia: A Case Report and Literature Review

**DOI:** 10.7759/cureus.56968

**Published:** 2024-03-26

**Authors:** Emmanuel A Agyemang, David M Makanga, Malaz Abdallah, Frances Ogunnaya, Shari Forbes

**Affiliations:** 1 Internal Medicine, Newark Beth Israel Medical Center, Newark, USA

**Keywords:** t-cell immunity, hiv seronegative, cd4+ lymphopenia, idiopathic cd4 lymphocytopenia, idiopathic cd4+ lymphocytopenia

## Abstract

Idiopathic CD4 lymphocytopenia (ICL) is a rare condition where CD4 T cell counts are low, similar to advanced human immunodeficiency virus (HIV) infection but without acquired immunodeficiency syndrome (AIDS)-related symptoms. The cause is unknown, and theories suggest issues with T cell production, survival, migration, or immune system dysregulation. Diagnosis involves ruling out other causes of low CD4 T cells. Treatment is based on managing infections and may include immunomodulatory therapies, but evidence is limited. Clinical presentations vary widely, including infections, autoimmune disorders, and malignancies. This study explores challenges in diagnosing persistent fevers and lymphopenia, the role of medical history in treatment, HIV screening issues, UTI management in recurrent cases, and the importance of follow-up care for unresolved symptoms or abnormal lab results.

This study utilized a case study approach, focusing on the detailed presentation, evaluation, and management of the patient. Data were collected from the patient's medical records, including laboratory tests. Relevant literature was reviewed to provide context and support for the discussion of diagnostic challenges and management strategies.

This case highlights the importance of considering uncommon presentations of common infections in patients with complex medical histories. It underscores the need for thorough evaluation, including comprehensive medical history, diagnostic testing, and follow-up care, to ensure accurate diagnosis and appropriate management. By sharing this case, we aim to enhance the awareness and understanding of such presentations among healthcare providers, leading to improved patient care and outcomes.

## Introduction

Idiopathic CD4 lymphocytopenia (ICL) is a rare condition characterized by a persistent and unexplained decrease in circulating CD4 T lymphocytes, the immune cells that play a central role in orchestrating the immune response against pathogens. First described in the early 1990s, ICL is distinguished by the fact that it occurs in the absence of human immunodeficiency virus (HIV) infection or any other known immunodeficiency disorder [1]. The exact cause of ICL remains unknown, and the condition poses significant challenges in terms of diagnosis and management. Patients with ICL typically present with low CD4 T cell counts, similar to those seen in advanced HIV infection, yet they do not exhibit the characteristic signs and symptoms of acquired immunodeficiency syndrome (AIDS), such as opportunistic infections or certain types of cancers [2].

Several hypotheses have been proposed to explain the pathogenesis of ICL. These include abnormalities in T cell production or survival, defects in T cell migration or homing, and dysregulation of the immune system, leading to increased T cell destruction. However, none of these theories has been conclusively proven, highlighting the need for further research into the underlying mechanisms of this enigmatic condition. The diagnosis of ICL is based on the exclusion of other causes of CD4 lymphocytopenia such as HIV infection, autoimmune disorders, and certain medications. It is essential to carefully evaluate patients with unexplained CD4 T cell depletion to rule out these other potential etiologies [3]. Given the rarity of ICL, there is limited evidence regarding its optimal management. Treatment strategies are predominantly empirical, focusing on preventing and managing opportunistic infections, mirroring the approach employed in individuals with AIDS. Immunomodulatory interventions, like interleukin-2 (IL-2) or antiretroviral therapy (ART), have been utilized in select cases, yet their effectiveness remains ambiguous [3]. Affected individuals may have a wide variety of clinical presentations, ranging from infections to autoimmune disorders or even malignancy.

## Case presentation

A 44-year-old Haitian male with a past medical history of completely treated syphilis presented to the ED with complaints of fever and pain on micturition. He reported he has been having painful urination for the past seven months, which has persisted despite completing treatment with some medications prescribed to him by his physicians in New York. He was in this state of health until 10 days before presentation when he started experiencing persistent high fevers that were present throughout the day, relapsed a few hours after taking ibuprofen, and was associated with chills, increased frequency of urination, foul-smelling urine, and occasional gross hematuria. He denied any abdominal pains, nausea, vomiting, neck stiffness, or headaches. On initial evaluation at the ED, the patient was febrile (102.7 °F), and his lab revealed leukocytosis (WBC 21.5) with neutrophilic predominance (90%), elevated creatinine, turbid-appearing urine, and 3+ leukocyte esterase, blood, and protein respectively on urinalysis. Given his past medical and sexual history, an HIV screen was performed which was reactive. Upon further inquiry about the patient's knowledge of his HIV status and previous antiretroviral medication use, he denied knowledge and requested repeat testing. Subsequent enzyme-linked immunoassay (ELISA) tests were non-reactive, HIV ½ differentiation was negative, and his viral load was undetectable. Of note, his T cell subset panel was remarkable for a total lymphocyte count of 518.2 (1080-4840), CD4 T cell 227.4 (356-2856), and CD8 T cell 75.1 (162-1888). His urine cultures grew Escherichia coli, which was sensitive to piperacillin-tazobactam with a resolution of his febrile and urinary symptoms after three days of IV treatment. The patient was discharged home on seven days of oral trimethoprim-sulfamethoxazole, with an outpatient follow-up appointment in two months. A repeat laboratory test done at the clinic showed a persistently low CD4 T-cell (251), and the patient was counseled about his lymphopenic state.

## Discussion

T cells play a crucial role in the immune response against infections. They are a type of white blood cell that identifies and destroys infected cells, including those infected by bacteria, viruses, and fungi. T cells also play a role in coordinating the overall immune response by activating other immune cells and producing cytokines, which are signaling molecules that help regulate the immune response [4]. CD4 is a glycoprotein found on the surface of T-helper cells, a subset of T cells that plays a central role in orchestrating the immune response. CD4 acts as a co-receptor for the T cell receptor (TCR), helping bind antigens presented by antigen-presenting cells (APCs) and initiating the activation of T cells [5]. CD4 T cells are essential for the activation of other immune cells, including cytotoxic T cells and B cells, which are responsible for killing infected cells and producing antibodies, respectively [6]. The normal range for CD4 T cell counts in healthy individuals is typically between 500 and 1500 cells/mm^3^ of blood. When the CD4 count falls below 500 cells/mm^3^, individuals are considered to have a weakened immune system and are at greater risk for opportunistic infections, which are infections that occur more frequently or are more severe in individuals with weakened immune systems [7]. In summary, T cells, including CD4 T cells, play a critical role in mediating the immune response against infections. A decrease in CD4 T cell count below 500 cells/mm^3^ increases the risk of opportunistic infections, highlighting the importance of these cells in maintaining immune function.

The association between low CD4 T cell levels and HIV/AIDS is well-established, as HIV specifically targets and destroys CD4 T cells, leading to a weakened immune system and increased susceptibility to infections. However, cases of unexplained low CD4 counts in HIV-seronegative patients, now known as ICL, were first reported in 1989 [8]. It was not until 1992 that the US Center for Disease Control and Prevention (CDC) defined ICL as a clinical condition characterized by a documented absolute CD4 T-lymphocyte count of less than 300 cells/mm^3^ or less than 20% of total T cells on two separate occasions at least six weeks apart. Importantly, these low CD4 counts occur without evidence of HIV-1 or HIV-2 infection and in the absence of any other known immunodeficiency or therapy that could decrease CD4 T cell levels [2]. The recognition and definition of ICL by the CDC were significant milestones in understanding and categorizing this rare immune disorder. Despite the similarities in low CD4 counts, ICL differs from HIV/AIDS in that patients with ICL do not exhibit the typical signs and symptoms of AIDS such as opportunistic infections or certain types of cancers associated with advanced HIV infection [9]. The etiology of ICL remains unclear, and the condition presents challenges in terms of diagnosis and management. Further research is needed to elucidate the underlying mechanisms of ICL and to develop more effective strategies for its diagnosis and treatment.

Most cases of ICL are diagnosed in adulthood, with a mean age of presentation ranging from 17 to 78 years. There appears to be a slight male predominance, with a male-to-female ratio of 1.6:1 based on one study involving 47 patients with ICL [10]. Since its official definition by the US Center for Disease Control and Prevention (CDC) in 1992, the exact cause of ICL has remained unclear. While some rare familial cases have been reported, suggesting a potential genetic or hereditary component, most cases occur sporadically [11]. Current theories regarding the etiology of ICL suggest that it may result from dysregulation of the immune system, leading to the development of antibodies against T cells. Another hypothesis proposes that ICL may be due to increased T cell apoptosis (cell death), decreased production of T cell precursors, or decreased cytokine production, which are all essential for the maintenance of normal immune function [12]. The complex and multifactorial nature of ICL underscores the need for further research to better understand its underlying mechanisms. An improved understanding of the etiology of ICL could lead to more effective diagnostic strategies and potentially targeted treatments for this rare and poorly understood immune disorder.

ICL is a heterogeneous condition, meaning that it can manifest in a variety of clinical presentations, ranging from asymptomatic to life-threatening opportunistic infections, autoimmune diseases, or malignancies. Infections are the most common manifestation of ICL and can include a wide range of pathogens such as cryptococcosis, candidiasis, tuberculosis, toxoplasmosis, human papillomavirus, herpes viruses, and Pneumocystis jiroveci. These infections are typically seen in individuals with compromised immune systems, highlighting the critical role of CD4 T cells in combating these pathogens [13]. In addition to infections, ICL can also be associated with autoimmune conditions, which occur when the immune system mistakenly attacks the body's tissues. Autoimmune conditions that have been reported in association with ICL include systemic lupus erythematosus, sarcoidosis, autoimmune hemolytic anemia, Sjögren's syndrome, and psoriasis. The underlying mechanisms linking ICL to autoimmune diseases are not fully understood but may involve dysregulation of the immune system [14].

Furthermore, individuals with ICL may also be at increased risk for certain malignancies, particularly lymphomas and squamous cell carcinomas. The exact relationship between ICL and malignancy is unclear, but it is thought to be related to the underlying immune dysfunction seen in ICL [15]. Overall, the diverse clinical manifestations of ICL underscore the importance of considering this condition in the differential diagnosis of patients presenting with unexplained CD4 lymphocytopenia, particularly when they have a history of recurrent or unusual infections, autoimmune diseases, or malignancies. Prompt recognition and management of ICL are essential to prevent complications and improve outcomes for affected individuals.

Despite the various complications associated with ICL, there are currently no approved therapies specifically targeted for this condition. However, the management of infectious complications in patients with ICL is largely based on guidelines established for HIV/AIDS patients with low CD4+ T-lymphocyte counts [16].

Because the opportunistic infections that affect patients with ICL are similar to those seen in HIV-positive individuals with low CD4+ T cell counts, antimicrobial therapy is often used to manage and prevent these infections in patients with ICL. Depending on the clinical presentation, antimicrobial therapy is typically guided by the specific pathogens involved and may include antifungal, antibacterial, and antiviral agents [17]. In addition to antimicrobial therapy, supportive care measures, such as vaccinations, prophylactic antibiotics, and close monitoring for signs of infection, are important components of managing patients with ICL. Regular monitoring of CD4+ T cell counts and overall immune function can help guide treatment decisions and assess response to therapy [17]. While there is currently no specific targeted therapy for ICL, ongoing research is aimed at better understanding the underlying mechanisms of the condition and developing more effective treatment strategies. Until then, management of ICL will continue to focus on the prevention and treatment of infectious complications, similar to the approach used in HIV/AIDS patients.

## Conclusions

Idiopathic CD4 lymphopenia (ICL) is a rare immunodeficiency disorder of unknown cause. It is characterized by low levels of CD4 T cells, which makes individuals susceptible to opportunistic infections, autoimmune diseases, and malignancies. There is currently no cure for ICL, but treatment focuses on managing and preventing infections based on treatment protocols for similar patients with HIV seropositivity.
